# Dispersion of single-walled carbon nanotubes modified with poly-l-tyrosine in water

**DOI:** 10.1186/1556-276X-6-128

**Published:** 2011-02-10

**Authors:** Mio Kojima, Tomoka Chiba, Junichiro Niishima, Toshiaki Higashi, Takahiro Fukuda, Yoshikata Nakajima, Shunji Kurosu, Tatsuro Hanajiri, Koji Ishii, Toru Maekawa, Akira Inoue

**Affiliations:** 1 Bio-Nano Electronics Research Centre, Toyo University 2100, Kujirai, Kawagoe, Saitama 350-8585, Japan; 2 Faculty of Life Sciences, Toyo University 1-1-1 Izumino, Itakura-machi, Oura-gun, Gunma 374-0113, Japan; 3 Graduate School of Life Sciences, Toyo University, 1-1-1 Izumino, Itakura-machi, Oura-gun, Gunma 374-0113, Japan; 4 Graduate School of Interdisciplinary New Science, Toyo University, 2100, Kujirai, Kawagoe, Saitama 350-8585, Japan; 5 Asylum Technology Co. Ltd., 3-20-12 Yushima, Bunkyo-ku, Tokyo 113-0034, Japan

## Abstract

In this study, complexes composed of poly-l-tyrosine (pLT) and single-walled carbon nanotubes (SWCNTs) were produced and the dispersibility of the pLT/SWCNT complexes in water by measuring the *ζ *potential of the complexes and the turbidity of the solution were investigated. It is found that the absolute value of the *ζ *potential of the pLT/SWCNT complexes is as high as that of SWCNTs modified with double-stranded DNA (dsDNA) and that the complexes remain stably dispersed in the water at least for two weeks. Thermogravimetry analysis (TGA) and visualization of the surface structures of pLT/SWCNT complexes using an atomic force microscope (AFM) were also carried out.

## 

Carbon nanotubes (CNTs) are promising functional nanomaterials, which may initiate new industries in the twenty-first century, thanks to their unique properties, such as extremely high thermal conductivity and mechanical strength, conducting or semiconducting characteristics depending on their chirality, and so on and, therefore, there is a variety of possible applications of CNTs to a wide range of areas including bio-related ones: e.g., the development of biosensing, bioelectrochemical and biomedical devices, and drug delivery systems [1]. However, one serious problem arises when CNTs are used in bio-related fields, that is, their poor dispersibility in water. There have been quite a few studies aiming at improving the dispersibility of CNTs in water by attaching foreign molecules, such as DNA [2-6], proteins [7-10], polymers [11-18], surfactants [19-22], and other compounds [23-26] to CNTs. Wang et al. used poly-l-lysine for the improvement of the dispersibility of CNTs and investigated the effects of the pH and temperature on the dispersion of poly-l-lysine/single-walled carbon nanotube (pLL/SWCNT) complexes in water [27]. The pLL/SWCNT complexes showed reversible changes in dispersibility against the pH of the water, that is, the complexes dispersed stably in the case of pH < 9, whereas they coagulated to form clusters or bundles in the case of pH > 9. Li et al. investigated the effect of the surface modification of oxidized multi-walled carbon nanotubes (MWCNTs) with pLL on the dispersibility of the complexes and showed that the pLL/MWCNT complexes remained stably dispersed in water for at least 10 days [28]. Cousins et al. showed that *N*-(fluorenyl-9-methoxycarbonyl)/tyrosine/CNT (Fmoc/Tyr/CNT) complexes dispersed stably in water [29]. In this article, focus is laid on the creation of poly-l-tyrosine (pLT)/SWCNT complexes, supposing that pLT can be adsorbed onto SWCNTs via the interactions among six-membered rings [29], and the dispersibility of the complexes in water is investigated, which has not so far been carried out. The surfaces of SWCNTs with pLT have been modified and the dispersibility of the complexes by measuring the *ζ *potential of pLT/SWCNT complexes and the turbidity of the solution were evaluated. Thermogravimetry analysis (TGA) and visualization of the surface structures of pLT/SWCNT complexes using an atomic force microscope (AFM) were then carried out.

First of all, the authors produced pLT/SWCNT complexes. The SWCNTs were brayed, the average diameter and length of which were, respectively, 2 nm and 10 μm (Shenzhen Nanotech Port Co., Ltd., Baoan, Shenzhen, China), in an agate mortar and then dispersed in distilled water (DW). The mass concentration of SWCNTs was 1.0 mg ml^-1^. The SWCNTs solution was sonicated using an ultrasonic cleaner (W-113 Ultrasonic multi cleaner, Honda Electronics Co., Ltd., Aichi, Japan) at 4°C for 8 h. The pLT (MP Biomedicals, LLC, Solon, OH, USA), the molecular weight of which varied from 12,000 to 35,000, was dissolved in 0.02 M KOH aqueous solution. The mass concentration of pLT was 1.0 mg ml^-1^. The SWCNTs and pLT solutions were mixed together at 30°C by shaking them at a frequency of 20 Hz by a shaker (Bio-Shaker BR-40LF, Taitec Co., Ltd., Aichi, Japan) for 1 h. After incubation, the pLT and SWCNT solution mixture was centrifuged at 1.5 × 10^4 ^rpm at 4°C for 30 min using a micro-centrifuge (MX-301, Tomy Seiko Co., Ltd., Tokyo, Japan), and then the supernatant was removed. The same volume of distilled water as that of the removed supernatant was added to the pLT/SWCNT solution, and this procedure was repeated three times so that any pLT, which had not been adsorbed earlier onto SWCNTs, was removed. The *ζ *potential of pLT/SWCNT complexes was measured using the dynamic scattering method (Zetasizer nano-zs, Malvern Instruments Ltd., Worcestershire, UK). The turbidity of the pLT/SWCNT complex solution was also measured using a spectral photometer (U-2001 Spectrophotometer, Hitachi High-Technologies Corp., Tokyo, Japan). Note that the normalized turbidity is defined as follows: log_10_(*I*_in_/*I*_out_)_t_/log_10_(*I*_in_/*I*_out_)_*t*=0_, where *I*_in _and *I*_out _are, respectively, the powers of the incident and transmitted laser beams of 600-nm wavelength, and the turbidity at time *t *is normalized by that at the initial time *t *= 0. To compare the *ζ *potential and turbidity of the present pLT/SWCNT complexes with those of other materials, the following solutions were also prepared: (1) SWCNTs dispersed in DW (0.1 mg ml^-1^), (2) SWCNTs dispersed in 0.1% Polyoxyethylene(10)Octylphenyl Ether (TritonX-100) (Wako Pure Chemical Industries, Ltd., Osaka, Japan) solution, and (3) double-stranded DNA (dsDNA)/SWCNT complexes dispersed in DW. See Appendix for the preparation of SWCNTs dispersed in TritonX-100 solution and the production of the dsDNA/SWCNT complexes. The thermogravimetric analysis (TGA) (DTG-60H, Shimadzu Corp., Tokyo, Japan) at a heating rate of 5 K min^-1 ^from 20°C up to 800°C in nitrogen was carried out. The surface structures of pLT and pLT/SWCNT complexes using an atomic force microscope (AFM) were also visualized (MFP-3D, Asylum Research Co., Santa Barbara, CA, USA) through an ac non-contact mode by putting a drop each of pLT and pLT/SWCNT solutions onto a silicon substrate and then evaporating them naturally.

Figure 1a shows the sedimentation of SWCNTs in DW and the dispersion of pLT/SWCNT complexes in DW. Note that the mass concentrations of SWCNTs were 0.1 mg ml^-1 ^in both cases. The pLT/SWCNT complexes remained stably dispersed in DW for at least 14 days at 25°C, whereas the SWCNTs without any surface modification gradually coagulated to each other, and finally, were completely sedimented in DW (see also Figure 1c). Figure 1b shows the *ζ *potential of each material: i.e. (a) SWCNTs in DW, (b) SWCNTs in TritonX-100 solution, (c) dsDNA/SWCNT complexes in DW, and (d) pLT/SWCNT complexes in DW. Note that the data shown in Figure 1b were taken 14 days after each material had been dispersed in DW. The absolute value of the *ζ *potential of pLT/SWCNT complexes in DW was higher than that of SWCNTs in TritonX-100 solution and was slightly lower than that of dsDNA/SWCNT complexes in DW. It is known that the particles disperse stably in water when the absolute value of the *ζ *potential of each particle is greater than 20 mV [30,31], with which the present results coincide: that is, for the pLT/SWCNT and dsDNA/SWCNT complexes, the absolute values of the *ζ *potentials were, respectively, 42.3 and 46.2 mV, and the complexes remained stably dispersed in DW for al least 14 days: however, SWCNTs in DW, the absolute value of the *ζ *potentials of which was 13.0 mV, finally got sedimented (see also Figure 1a). SWCNTs in TritonX-100 solution, the absolute value of the *ζ *potential of which was 30.0 mV, also got dispersed stably. The time variation of the turbidity of each solution is shown in Figure 1c. The turbidity of the pLT/SWCNT in DW, which was slightly lower than that of the dsDNA/SWCNT in DW, but higher than that of the SWCNTs in TritonX-100 solution, was almost constant for 14 days, whereas the turbidity of the SWCNTs in DW immediately decreased due to quick coagulations and sedimentations of SWCNTs in DW. There is a clear correlation between the turbidity of each solution and the *ζ *potential of each material in the solution: that is, the higher the absolute value of the *ζ *potential of a material is, the higher the turbidity of the solution becomes (see Figure 1b, c). The TGA data obtained for pLT, SWCNTs, and pLT/SWCNTs complexes are shown in Figure 2. Both pLT and pLT/SWCNT complexes started decomposing at 300°C, whereas SWCNTs did not decompose at least up to 800°C. Note that the decomposition temperature of pLT obtained by the present TGA analysis, that is, 300°C, coincides with that measured previously [32]. pLT was definitely adsorbed onto SWCNTs judging by the TGA data of pLT/SWCNT complexes. The quantum calculations of the interactions between a single tyrosine molecule and a [6,6] SWCNT were carried out by the PM3 method (Gaussian03, Gaussian Co., Pittsburgh, PA, USA). As shown in Figure 3, a tyrosine molecule was adsorbed onto a SWCNT via the interactions between the six-membered rings as in the case of Fmoc/Tyr/SWCNT complexes [29]. The gap between the six-membered rings was approximately 0.45 nm, which is quite similar to that between graphitic layers [33]. It is supposed that the decomposition temperature of the pLT adsorbed onto SWCNTs was almost the same as that of pLT, that is, 300°C (see Figure 2), since the interactions between the rings are not very strong [29]. Judging by the weight loss obtained by the TGA analysis (Figure 2), 0.2 μg of pLT was adsorbed onto 1 μg of SWCNTs on average. AFM images of pLT and pLT/SWCNT complexes are shown in Figure 4. pLT without any immobilizations onto SWCNTs folded by itself to form sphere-like structures (see Figure 4a). The whole surfaces of the SWCNTs were covered with pLT, and the thickness of the pLT layers adsorbed onto SWCNTs varied from 1 to 4 nm (Figure 4b, c). It is supposed that pLT was adsorbed onto SWCNTs via the interactions between six-membered rings as mentioned above, and pLT folded to form sphere-like structures on the surfaces of SWCNTs, the thickness of which varied cyclically in the axial direction of the SWCNTs (Figure 4c). A TEM image of a pLT/SWCNT complex is also shown in the Additional file 1, where the mass concentration of pLT in DW was set at 0.2 mg ml^-1 ^to obtain a clearer image. The pLT/SWCNT complexes dispersed stably in water thanks to the polar -OH group in tyrosine. In the case of tryptophan/SWCNT complexes, on the other hand, they did not disperse in water due to the hydrophobic group in tryptophan although it was adsorbed onto SWCNTs via the interactions among six-membered rings. Biomolecules such as enzymes can be attached to pLT, and therefore enzyme/pLT/SWCNT complexes can be produced so that new biosensors and devices may be developed in combination with SWCNT electronics [34]. The authors will be carrying out spectroscopic analyses such as Raman and Infrared spectroscopies of pLT/SWCNT complexes so that the structures of and conformational changes in pLT immobilised on SWCNTs may be clearly understood. The authors will also be investigating the adsorption of various biomolecules, viruses, and bacteria onto pLT/SWCNT complexes so that the complexes may be used as adsorbers, filters, or screening devices for organic molecules, viruses, and bacteria. The authors will also be measuring the electric and electronic properties of the complexes so that the above mentioned biosensors may be developed.

**Figure 1:**
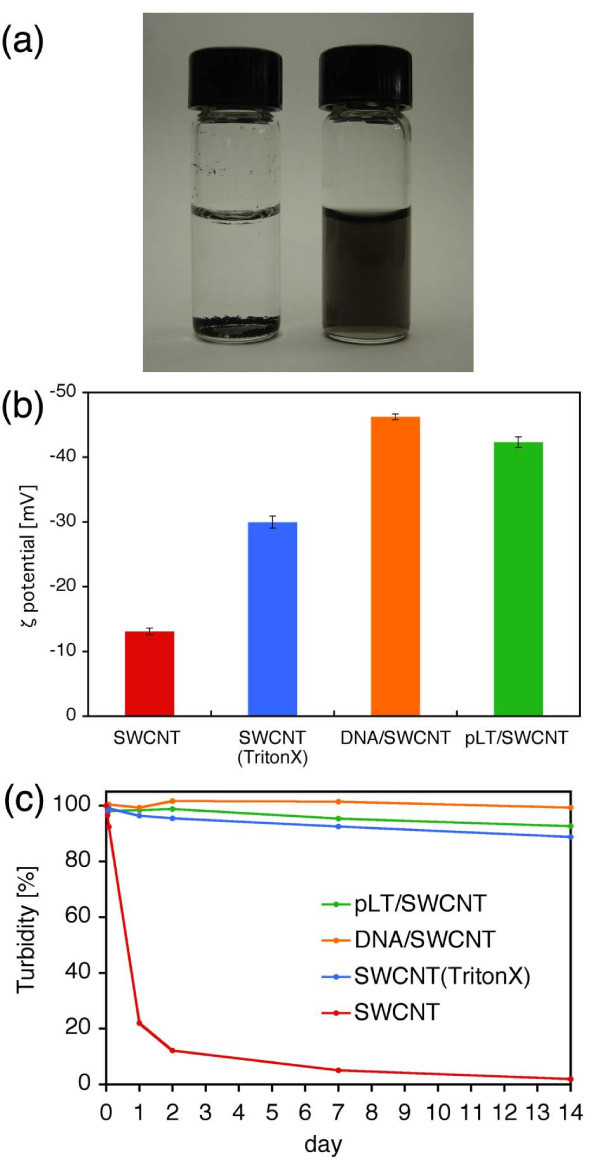
**Dispersion of SWCNTs in distilled water**. **(a) **Dispersion of SWCNTs in distilled water 14 days after the preparation. Left: SWCNTs without any surface modification. SWCNTs coagulated to each other and finally sedimented. Right: SWCNTs modified with pLT. pLT/SWCNT complexes remained stably dispersed for at least 14 days. **(b) ***ζ *potentials of SWCNTs in DW, SWCNTs in TritonX-100 solution, dsDNA/SWCNT complexes in DW, and pLT/SWCNT complexes in DW. The *ζ *potentials were measured 14 days after the preparation. The *ζ *potential of pLT/SWCNT complexes in DW is slightly lower than that of dsDNA/SWCNT complexes in DW, but higher than that of SWCNTs in TritonX-100 solution. **(c) **Time variations of the turbidity of SWCNTs in DW, SWCNTs in TritonX-100 solution, dsDNA/SWCNT complexes in DW, and pLT/SWCNT complexes in DW. The turbidity of SWCNTs in TritonX-100 solution, dsDNA/SWCNT complexes in DW, and pLT/SWCNT complexes in DW hardly changed for 14 days, whereas SWCNTs without any surface modification sedimented quickly. The turbidity of pLT/SWCNT complexes in DW was slightly lower than that of dsDNA/SWCNT complexes in DW, but higher than that of SWCNTs in TritonX-100 solution.

**Figure 2:**
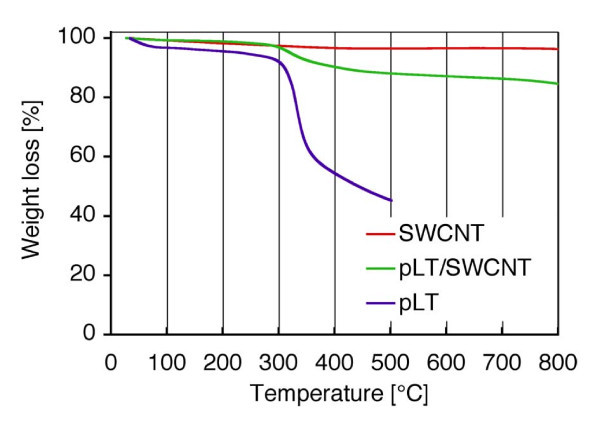
**TGA curves of SWCNTs, pLT, and pLT/SWCNT complexes**. pLT by itself and pLT adsorbed onto SWCNTs decomposed at 300°C, which suggests that pLT is adsorbed rather weakly onto the surfaces of the SWCNTs.

**Figure 3:**
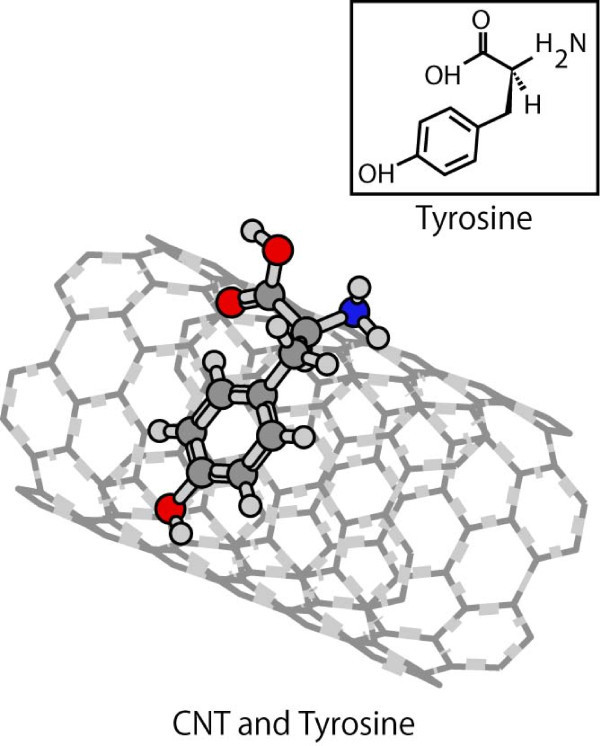
**Interaction between a single tyrosine molecule and a **[6,6]**SWCNT calculated by the PM3 method**. Tyrosine can be adsorbed onto the surface of SWCNT via the interactions among six-membered rings.

**Figure 4:**
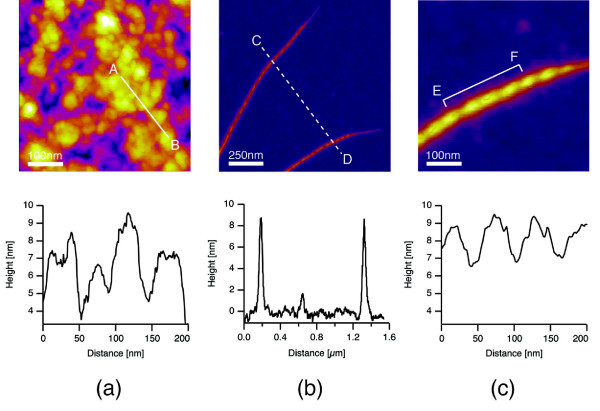
**AFM images of pLT and pLT/SWCNT complexes**. **(a) **AFM image of pLT and the height distribution along line A-B. PLT folded to form sphere-like structures on the surface of an Si substrate. **(b) **AFM image of pLT/SWCNT complexes and the height distribution along line C-D. **(c) **AFM image of a pLT/SWCNT complex and the height distribution along line E-F. The thicknesses of pLT adsorbed onto the SWCNT varied cyclically in the axial direction of the SWCNT.

In summary, pLT/SWCNT complexes were produced and it was found that the complexes dispersed stably in water, which coincided with the results of the measurement of the *ζ *potential of the complexes in DW and the turbidity of the pLT/SWCNT aqueous solution. The dispersibility of pLT/SWCNT complexes was as high as that of dsDNA/SWCNT complexes. pLT was adsorbed onto the SWCNTs via rather weak interactions among six-membered rings according to the TGA data, quantum calculations, and AFM images. AFM images showed that the surfaces of the SWCNTs were completely covered with pLT, and the thickness of the pLT on the SWCNTs varied cyclically in the axial direction. The result of this study suggests that any polypeptide, in which some aromatic amino acids are included, can be adsorbed onto SWCNTs via the interactions among six-membered rings and that the dispersibility and other physical and chemical properties of polypeptide/SWCNT complexes can be altered by choosing some appropriate amino acid sequence depending on the users' purposes.

## Abbreviations

AFM: atomic force microscope; dsDNA: double-stranded DNA; DW: distilled water; pLT: poly-l-tyrosine; SWCNTs: single-walled carbon nanotubes; TGA: thermogravimetry analysis.

## Competing interests

The authors declare that they have no competing interests.

## Authors' contributions

MK designed the study and carried out the experiment. TC participated in the dispersion experiment. JN participated in the dispersion experiment. TH performed AFM observation. TF carried out the TGA experiment and TEM observation. YN participated in the AFM observation. SK carried out the quantum calculation. TH participated in the design of the study. KI participated in the design of the study and AFM observation. TM participated in the design of the study, coordinated the study and wrote the manuscript. AI participated in the design of the study and coordinated the study. All authors read and approved the final manuscript.

## Appendix

Preparation of SWCNTs dispersed in TritonX-100 solution

0.1% TritonX-100 and 0.02 M KOH were mixed with 0.15 mg ml^-1 ^SWCNTs by a mixer (VORTEX-GENIE2, model G-560, Scientific Industries Inc., Bohemia, New York, USA) at 30°C for 1 h.

Production of dsDNA/SWCNT complexes

0.15 mg ml^-1 ^of SWCNTs and 0.5 mg ml^-1 ^of dsDNA were both dispersed in water. The above two solutions were mixed together by shaking them at 30°C at a frequency of 20 Hz for 1 h. After incubation, the dsDNA and SWCNT mixtures were centrifuged at 1.5 × 10^4 ^rpm at 4°C for 30 min, the supernatant was taken away, and DW was added to remove unadsorbed dsDNA molecules. The above washing procedure was repeated five times.

## Supplementary Material

Additional file 1 **TEM image of a pLT/SWCNT complex**. The mass concentration of pLT was set at 0.2 mg ml^-1 ^in DW to obtain a clearer image. Click here for file
