# Supplementary material for: Dispersion of single-walled carbon nanotubes modified with poly-l-tyrosine in water
Source: Nanoscale Res Lett. 2011 Feb 10;6(1):128. doi: 10.1186/1556-276X-6-128 (PMC3211174; doi:10.1186/1556-276X-6-128)

**Additional file 1**

Dispersion of single-walled carbon nanotubes modified with poly-L-tyrosine in water

Mio Kojima, Tomoka Chiba, Junichiro Niishima, Toshiaki Higashi,

Takahiro Fukuda, Yoshikata Nakajima, Shunji Kurosu, Tatsuro Hanajiri,

Koji Ishii, Toru Maekawa and Akira Inoue

TEM image of a pLT/SWCNT complex

The mass concentration of pLT was set at 0.2 mg ml^-1^ in DW to obtain a clearer image.


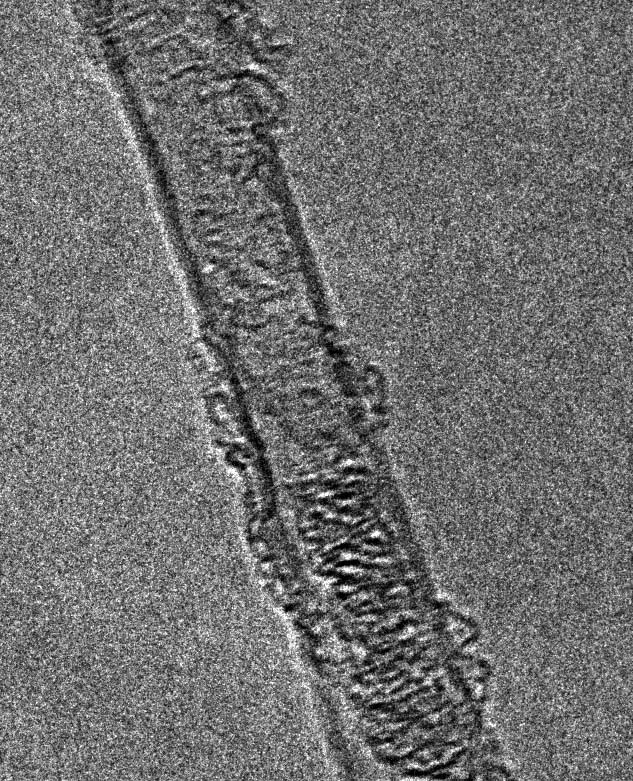

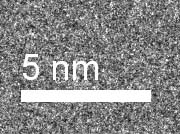

Supplement: Additional file 1 — TEM image of a pLT/SWCNT complex. The mass concentration of pLT was set at 0.2 mg ml-1 in DW to obtain a clearer image. [file 1556-276X-6-128-S1.DOC]
